# Remote Ischemic Conditioning Protects Diabetic Retinopathy in Streptozotocin-induced Diabetic Rats via Anti-Inflammation and Antioxidation

**DOI:** 10.14336/AD.2018.0711

**Published:** 2018-12-04

**Authors:** Changhong Ren, Hang Wu, Dongjie Li, Yong Yang, Yuan Gao, Yunneng Jizhang, Dachuan Liu, Xunming Ji, Xuxiang Zhang

**Affiliations:** ^1^Beijing Key Laboratory of Hypoxia Conditioning Translational Medicine, Beijing, China.; ^2^Department of Ophthalmology, Xuanwu Hospital, Capital Medical University, Beijing, China.; ^3^Department of Herbal Formula Science Medicine, Chinese Medicine College, Beijing University of Chinese Medicine, Beijing, China.; ^4^Center of Cerebrovascular Disease Research, University of Pittsburgh School of Medicine, Pittsburgh, USA.; ^5^Center of Stroke, Beijing Institute for Brain Disorder, Beijing 100069, China

**Keywords:** diabetes mellitus, diabetic retinopathy, limb remote ischemic conditioning, oxidative stress

## Abstract

Ischemic conditioning inhibits oxidative stress and inflammatory response in diabetes. However, whether limb remote ischemic conditioning (LRIC) has beneficial effects on diabetic retinopathy (DR) remains unknown. This study aims to investigate the protective effects of LRIC in retinal ganglion cell in streptozotocin (STZ) induced Type 1 diabetic rats. A total of 48 healthy male Sprague-Dawley (200-220g) rats were randomly assigned to the normal group, normal+LRIC group, diabetes mellitus (DM) group and DM+LRIC group. Streptozotocin (STZ, 60 mg/kg) was intraperitoneally injected into the rats to establish the diabetic model. LRIC was conducted by tightening a tourniquet around the upper thigh and releasing for three cycles daily (10 mins x 3 cycles). Retinas were harvested after 12 weeks of LRIC treatment for histopathologic, Western blot and ELISA analysis. Plasma were collected at the same time for ELISA analysis. LRIC alleviated diabetic retinopathy symptoms as evidenced by the increased number of retinal ganglion cells (P<0.01) and decreased glial fibrillary acidic protein (GFAP) expression level (P<0.01) in the rat retina. LRIC in DM rats exhibited anti-inflammatory and antioxidative effects as confirmed by the down-regulation of pro-inflammatory cytokine: interleukin-6 (IL-6), and the up-regulation of antioxidants: superoxide dismutase (SOD), and glutathione (GSH)/oxidized glutathione (GSSG). Furthermore, LRIC significantly downregulated VEGF protein expression in the retina (P<0.01). These results suggest that the antioxidative and anti-inflammatory activities of LRIC may be important mechanisms involved in the protective effect of LRIC in STZ-induced diabetic rats.

Diabetic retinopathy (DR), a leading cause of blindness, is one of the complications associated with diabetes [1]. increasing evidence support the idea that retinal neurons undergo degeneration, which may be part of vision impairment in the early stage of diabetes. In particular, retinal ganglion cells, which are predominantly responsible for conducting visual impulses to the brain [2]. However, effective neuroprotective agents are limited in preventing retinal neurons death in DR [2]. Currently, the most effective therapeutic options such as laser photocoagulation, corticosteroids, anti-vascular endothelial growth factor (anti-VEGF) agents and vitrectomy, are limited due to their considerable side e?ects (e.g. glaucoma) [3, 4]. Therefore, developing novel, mechanism-based therapeutic strategies is highly desirable for the clinical management of DR patients [5].

Hyperglycemia-mediated oxidative stress and in?ammation play important roles in the pathogenesis of DR [3, 4,[6]]. Shifting the delicate balance between the production and elimination of oxygen radicals or in?ammatory cytokines can result in cellular damage. Long-term hyperglycemia induces the overproduction of reactive oxygen species (ROS) that triggers oxidative stress. Low activities of antioxidative enzymes contribute to further accumulation of ROS. It has been reported that necrosis could cause oxidative stress-induced retinal cell death in turn promoting the expression of pro-in?ammatory genes. Upregulation of proin?ammatory cytokines such as TNF-α and interleukin-1β (IL-1β) were observed in the eyes of patients with DR [7]. TNF-α also upregulated the expression of VEGF in the retina in early stages of diabetes, which was associated with increased vascular permeability and disruption of the blood-retinal barrier [8, 9]. Thus, treatments that limit oxidative and in?ammatory e?ects could be highly beneficial for DR patients by reducing or preventing retinal complications.

Ischemic conditioning has been considered a form of protection against ischemic injury in a variety of organs through the initiation of endogenous protective mechanisms [10, 11]. Retinal ischemic conditioning induced by increasing intraocular pressure was reported to prevent and reduce retinal function impairment in both Type 1 and Type 2 diabetic animal models [12, 13]. However, there are significant limitations pertaining to local ischemic conditioning due to the characteristics and properties of inducing ischemic conditioning in vital organs such as the heart or brain. Many groups have demonstrated that limb remote ischemic conditioning (LRIC) has a neuroprotective effect in both experimental and clinical studies [14]. Brandli et al reported that LRIC had a protective effect by significantly increasing the amplitude of a- and b- waves in a light-induced retinal damage animal model [15]. LRIC twice daily could reduce sensitive C-reactive protein (CRP) and IL-6 in intracranial arterial stenosis patients [16]. Recently, we reported that repeated daily LRIC attenuated ischemia/reperfusion-induced retinal injury in rats through the increase in antioxidative defenses [17]. Taken together, the aim of the present study was to examine whether LRIC has therapeutic effects in DR prevention by modulating oxidative stress and inflammation.

## MATERIALS AND METHODS

### Animal models

48 healthy male Sprague-Dawley rats (240-250g) were used for this study. Type 1 DM was induced by a single injection of 1% streptozotocin (STZ, Sigma, Buchs, Switzerland) intraperitoneally (60 mg/kg freshly dissolved in citrate buffer, pH 4.2). The other groups received the same volume of citrate buffer (pH 4.2). After three days, rats with random blood glucose >16.7 mmol/L were included. Diabetic rats were randomly assigned to DM or DM+LRIC groups. Blood glucose level and weight for all rats were monitored every week.

### Limb remote ischemia conditioning protocol

**Figure 1.**
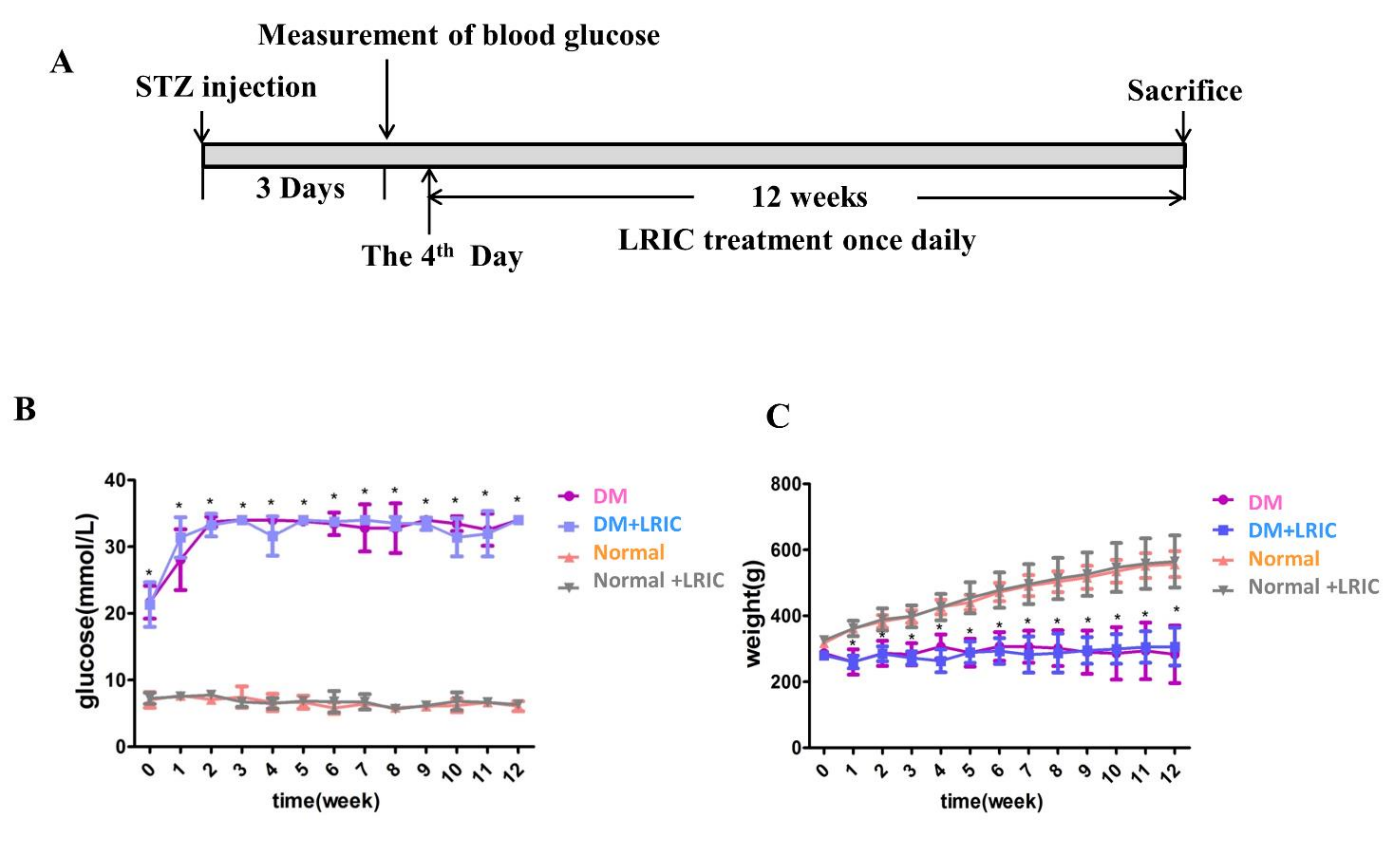
Effect of LRIC on blood glucose levels and body weight at 12 weeks after onset of diabetes. (A) Representative sketches of the experiment. (B) Quantification of blood glucose levels. (C) Quantification of body weight. Data are expressed as mean±SD, * P<0.05 (DM vs. Normal group). N=10 each group.

Remote ischemia preconditioning (LRIC) was conducted as previously described [17]. Rats were anesthetized with sodium pentobarbital (30 mg/kg) intraperitoneally. LRIC was conducted by tightening a tourniquet around the upper thigh and releasing for three cycles daily. Each cycle comprises 10 min of ischemia and 10 min of reperfusion. LRIC was applied at 4 days after STZ injection and then repeated every day thereafter for up to 12 weeks (Fig. 1A). Rats in the control and diabetic groups received sodium pentobarbital treatment alone. Anal temperature was maintained using heating pads. There were no negative effects on the function or tissue integrity of the limb [18, 19].

### Tissue processing

After anesthesia, rats were sacrificed and their eyes enucleated. The left retinas were isolated and stored at -80 °C. The right eyes were fixed in 4% paraformaldehyde for 4 hours at 4°C. The cornea and lens were removed, and eye cups were dehydrated and embedded in paraffin. The tissues were sectioned at a thickness of 3 μm along the horizontal meridian through the optic nerve head. Tissue sections were then stained with hematoxylin and eosin.

### Immunohistochemistry 

Tissue sections were deparaffinized and washed in 0.1 M sodium phosphate buffered saline (PBS) [20], then processed with 3% H_2_O_2_, incubated in 1% BSA for 1 hour and incubated with primary antibodies anti-Brn3a (1:100, Santa Cruz Biotechnology, CA) and anti-glial fibrillary acidic protein (GFAP, 1:100, Santa Cruz Biotechnology, CA) at 4°C overnight. After washing with PBS, sections were incubated with the appropriate secondary antibodies for 1 hour at room temperature. The area of the retina was measured with the computer program Image Pro Plus (IPP, Media Cybernetics) in a single-blind fashion by an independent investigator. GFAP staining was quantified from three images. The number of positive cells in the ganglion cell layer was measured at 200 μm away from the optic nerve head by an independent investigator. The average was then calculated and presented as the representative value for each group.

### Measurement of ROS production

The method for detecting ROS was performed as described previously [21]. Homogenized retinal samples taken from the rats were diluted to 10 mg/mL after measuring protein concentration using the bicinchoninic acid (BCA) method. After 30 minutes of incubation, H_2_O_2_ levels in brain homogenates were determined using 50 µmol/L Amplex red, 0.1 U/mL horseradish peroxidase, and respiratory substrates (4 mmol/L pyruvate, 2 mmol/L malate, 2 mmol/L glutamate, and 0.8 mmol/L complex V inhibitor oligomycin) at 37 °C on a DTX-880 Multimode Detector.

### Measurement of SOD and GSH/GSSG levels in plasma

Rats were anesthetized and sacrificed after 12 weeks of LRIC treatment. For SOD/catalase ratio and GSH/GSSG ratio assays, the retinas were sonicated in PBS (pH 7.4) for two 30 sec bursts. For glutathione quantification, the retinas were sonicated in 5% 5-sulfosalicylic acid. Total protein concentration was measured by the BCA Protein Assay kit reagent (Thermo Electron Corporation, USA). SOD and CAT activities were assayed using the SOD Assay Kit-WST (Dojindo Molecular Technologies, Gaithersburg, Maryland, USA) and Catalase Assay kit (Beyotime Biotechnology, Nanton, China), respectively. Enzyme activities were calculated as units/mg of protein and expressed as the relative value to normal control animals. The redox state of the whole body was determined as the ratio of GSH/GSSG using a GSH and GSSG Assay Kit (Beyotime Biotechnology, Nanton, China). The SOD/catalase ratio and GSH/GSSG ratio were expressed as ratios of their absolute values.

### Enzyme-linked immunosorbent assay (ELISA)

Rats were anesthetized and sacrificed after 12 weeks of LRIC treatment. The levels of VEGF, IL-6, TNF-α and IL-1β in rat retina and plasma were analyzed using ELISA according to the manufacturer’s protocol (R&D, USA). Fresh blood samples from the inferior vena cava were collected into a vacuum tube with sodium citrate (3.2% w/v) and centrifuged at a speed of 3500 rpm for 15 min at room temperature. Plasma were obtained and stored in -80 °C until further analysis [22].

### Western blot

Protein was isolated from the rat retina at 12 weeks after LRIC treatment. Protein (20 μg) was electrophoresed on 12.5% sodium dodecyl sulfate polyacrylamide gels then transferred to a polyvinylidene di?uoride membrane (Millipore Corporation, USA) [11]. Membranes were blocked for 1 h in 5% skim milk in 1xTris-buffered saline with 0.01% Tween-20 buffer and immersed overnight at 4 °C with the primary antibody against VEGF (1:1000; Abcam) [22]. GAPDH was used to verify equal protein loading. The specific reaction was visualized by the chemiluminescence substrate luminol reagent (GE Healthcare, UK). The optical density of protein was measured using Image-Pro Plus software 5.0 (Rockville, MD, USA) according to the manufacturer’s instructions. The mean amount of protein expression from the normal group was assigned a value of 1 to serve as reference.

### Statistical analysis

Data were expressed as mean ± standard deviation (SD) and were calculated using SPSS version 19.0 (SPSS, Chicago, IL, USA). Differences between groups were analyzed using One-way analysis of variance (ANOVA). Post hoc multiple comparisons were performed using Fisher’s LSD where appropriate. Comparisons of weight and blood glucose level across time points were analyzed by two-way repeated measures ANOVA. All analyses were performed with significance set at P<0.05.

## RESULT

### LRIC had no effect on body weight and blood glucose levels

Diabetes induced a significant increase of blood glucose level and significant reduction of body weight when compared to the control group (Fig. 1B, C). Treatment with LRIC had no effect on blood glucose level and body weight (Fig. 1B, C)

### LRIC treatment ameliorated Brn3a+ retinal ganglion cell loss in diabetic rats

**Figure 2.**
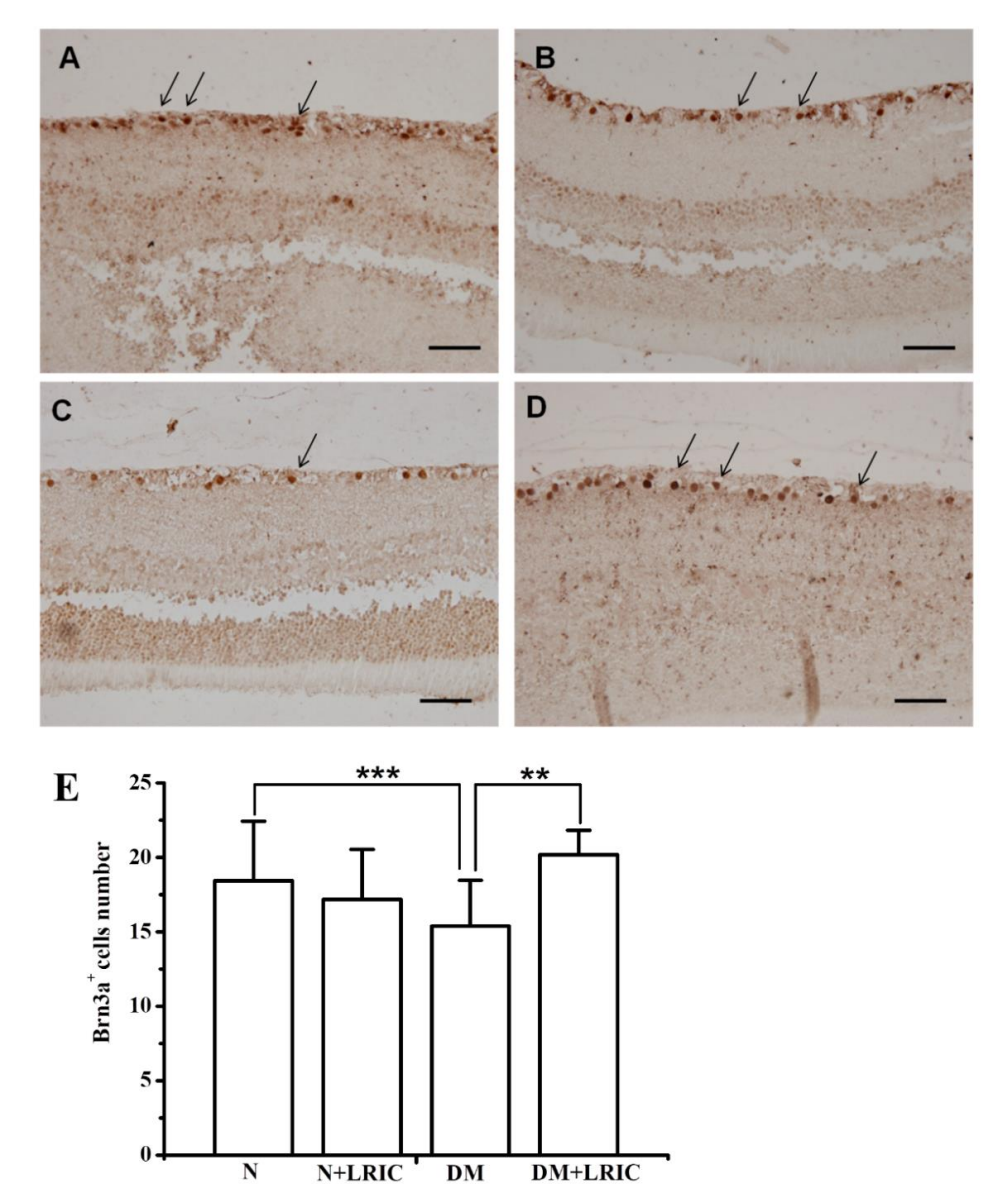
LRIC treatment ameliorated Brn3a^+^ retinal ganglion cell loss in diabetic rats. Immunohistochemical analysis of retinas of normal control group (A), normal+LRIC group (B), DM group (C) and DM+LRIC group (D). Arrows indicate Brn3a^+ ^RGCs, scale bar=50 μm. (E) Bar graphs depicting the average number of Brn3a^+^ RGCs in each group. Data are expressed as mean±SD, * *P*<0.05. N=5 each group.

To demonstrate RGC loss in our diabetic rat model, we quantitatively analyzed RGCs using the Brn3a marker. Brn3a+ cells were present in the ganglion cell layer and the nuclei of cells are predominantly stained (Fig. 2). The number of Brn3a+ cells in the RGC layer in the normal group, normal+LRIC group, DM group and DM+LRIC group are 18.45±3.97, 17.18±3.37, 15.41±3.07 and 20.18±1.64, respectively. Compared to normal control (Fig. 2A), there was a significant decrease of Brn3a+ cells in the DM group (P<0.05, Fig. 2C). The number of RGCs was significantly increased in the DM+LRIC group (Fig. 2D) compared with the DM group (P<0.01, Fig. 2E). Taken together, these data indicate that LRIC could prevent Brn3a+ RGCs loss in diabetic rats.

### LRIC treatment ameliorated retinal Müller cell activation in diabetic rats

**Figure 3.**
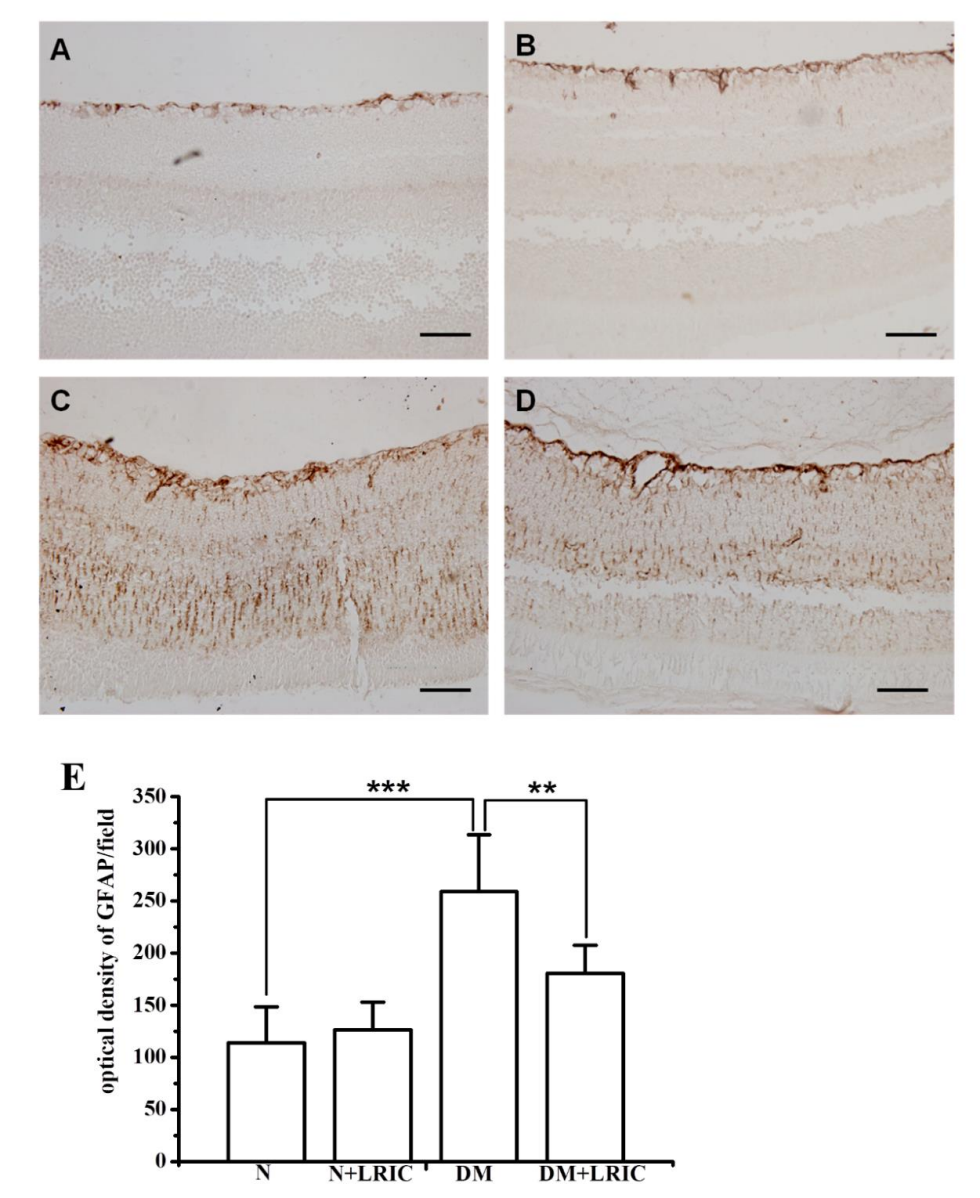
LRIC treatment ameliorated retinal Müller cell activation in diabetic rats. There was a significant increase in the level of GFAP expression in the diabetic retina (C) compared with the control retina (A). After 12 weeks of LRIC treatment, GFAP immunostaining decreased significantly in the diabetic retina (D). However, GFAP expression in the control retina was not affected by LRIC (B). Scale bar=50 μm. (E) Bar graphs depicting the the density of GFAP in each group. Data are expressed as mean±SD, ** *P*<0.01, *** *P*<0.001. N=5 each group.

To determine whether LRIC has beneficial effects on Müller cells, we also investigated the expression and localization of GFAP in rats’ retinas. GFAP immunostaining was performed to evaluate glial cell activation in the diabetic retina and LRIC. In normal rats, GFAP was expressed in the inner limiting membrane and retinal nerve fiber layer (Fig. 3A). In DM rats, however, GFAP expression was significantly increased and distributed throughout the retinas (Fig. 3C). However, GFAP staining was significantly reduced in DM+LRIC rats as compared with DM rats at 12 weeks after LRIC treatment (Fig. 3D, E).

### LRIC treatment attenuated oxidative stress induced by hyperglycemia

**Figure 4.**
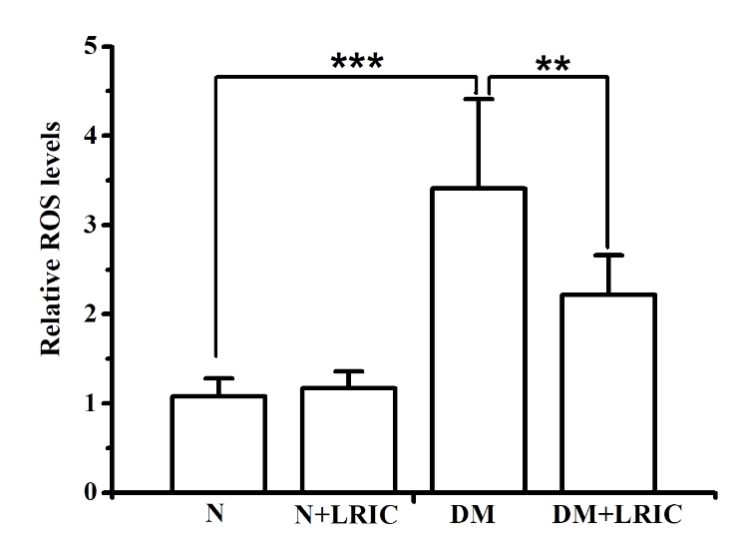
LRIC treatment attenuated oxidative stress induced by hyperglycemia. ROS production in the retina was evaluated at 12 weeks after LRIC treatment. The graph showed the relative reactive oxygen species levels in each group. *P<0.05, **P<0.01, N=7 each group.

To determine whether LRIC treatment can attenuate oxidative stress induced by hyperglycemia, ROS production in the retina was evaluated at 12 weeks after LRIC treatment. In DM rats, ROS levels were significantly increased compared with normal rats (P<0.01, Fig. 4). LRIC treatment induced a significant (P<0.01) reduction in ROS levels, suggesting the attenuation of oxidative damage.

### LRIC altered antioxidant enzyme levels in diabetic rats

**Figure 5.**
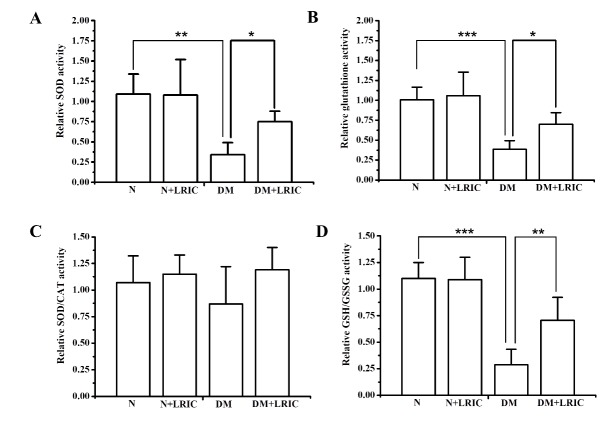
LRIC altered antioxidant enzyme levels in diabetic rats. Antioxidant enzyme levels in the retina were analyzed at 12 weeks after LRIC treatment. (A) Analysis of SOD activity. Data represent mean±SD of six independent experiments. (B) The analysis of total glutathione level. Data represent mean±SD of three independent experiments. (C) Analysis of SOD/CAT ratio. (D) Analysis of GSH/GSSG ratio. Data represent mean±SD of six independent experiments. *P< 0.05, ** *P*<0.01, N=7 each group.

SOD, catalase, and glutathione play important roles in the detoxification of ROS in the retina. To investigate the possible influence of LRIC treatment on antioxidant enzymes, we evaluated the activity of SOD, SOD/ catalase ratio, and glutathione levels at 12 weeks after LRIC treatment. Compared with normal control rats, retinal SOD and glutathione activities in DM rats were markedly decreased by 16.8% (P<0.05) and 81.5% (P<0.01), respectively (Fig. 5A, B). LRIC treatment significantly increased SOD activity by 19.1% (P < 0.05 versus the diabetic control group, Fig. 5).

However, LRIC had no effect on the glutathione and SOD/catalase ratio (Fig. 5B, C). The systemic redox status of the retina was estimated by determining the ratio of reduced glutathione (GSH)/oxidized glutathione (GSSG). The ratio of GSH/GSSG in DM rats was significantly lower compared with normal rats (P<0.001) (Fig. 5D). LRIC treatment significantly increased the ratio of GSH/GSSG by 19.1% (P<0.01 versus the diabetic control group, Fig. 5D).

### LRIC treatment attenuated retinal inflammation in diabetic rats

**Figure 6.**
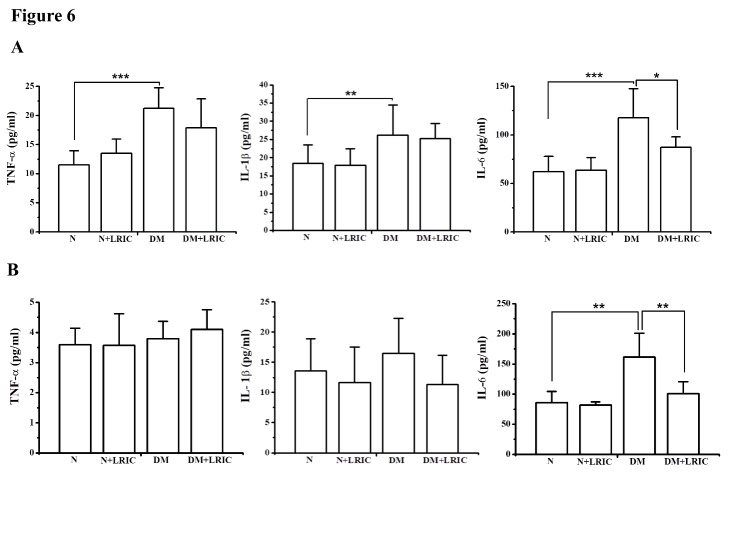
LRIC treatment attenuated retinal inflammation in diabetic rats. The inflammatory markers were analyzed in the diabetic rat retina and plasma at 12 weeks after LRIC treatment by using ELISA. (A) Inflammatory cytokines TNF-α, IL-1β and IL-6 levels in the rat retina. (B) Inflammatory cytokines TNF-α, IL-1β, IL-6 levels in the rat plasma. Data represent mean±SD of six independent experiments. *P<0.05, **P<0.01, N=7 each group.

Next, we examined the effect of LRIC on inflammatory markers in the diabetic rat retina and plasma at 12 weeks after LRIC treatment. The levels of IL-6, TNF-α and IL-1β in the retina were analyzed using ELISA. Compared with the normal control rat group, the levels of IL-6, TNF-α and IL-1β were markedly increased in the DM control rat group (P<0.05, Fig. 6). LRIC treatment significantly decreased the levels of IL-6 and TNF-α in the rat retina (P<0.05, Fig. 6). LRIC treatment also significantly suppressed the level of IL-6 in the plasma (P<0.05, Fig. 6).

### LRIC treatment decreased the level of VEGF in diabetic rats

**Figure 7.**
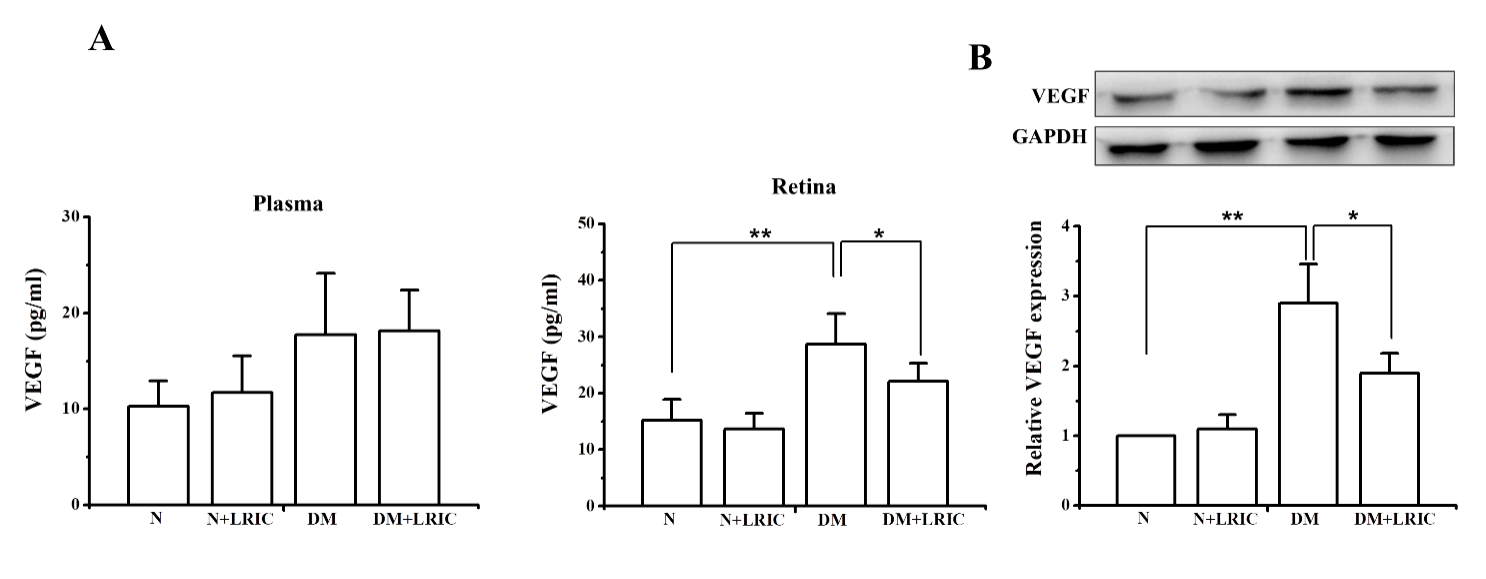
LRIC treatment decreased the level of VEGF in diabetic rats. The expression of VEGF was analyzed in the diabetic rat retina and plasma at 12 weeks after LRIC treatment. (A) The expression of VEGF in retinas was detected by ELISA for the di?erent groups. (B) The expression of VEGF in retinas was detected by Western blot for the di?erent groups. Data represent mean±SD of six independent experiments. *P<0.05, **P<0.01, N=7 each group.

Dysregulated retinal VEGF production during DR is one of the most devastating responses to oxidative stress and inflammation [22, 23]. First, we examined the VEGF protein level in plasma at 12 weeks after LRIC treatment. There was no significantly difference among the four groups (Fig. 7A). VEGF protein levels in the retina were determined using Western blotting and ELISA at 12 weeks after LRIC treatment. Compared with the normal control group, VEGF protein level was significantly increased in the retinas of DM control rats. However, LRIC treatment significantly attenuated this upregulation (Fig. 7).

## DISCUSSION

**Figure 8.**
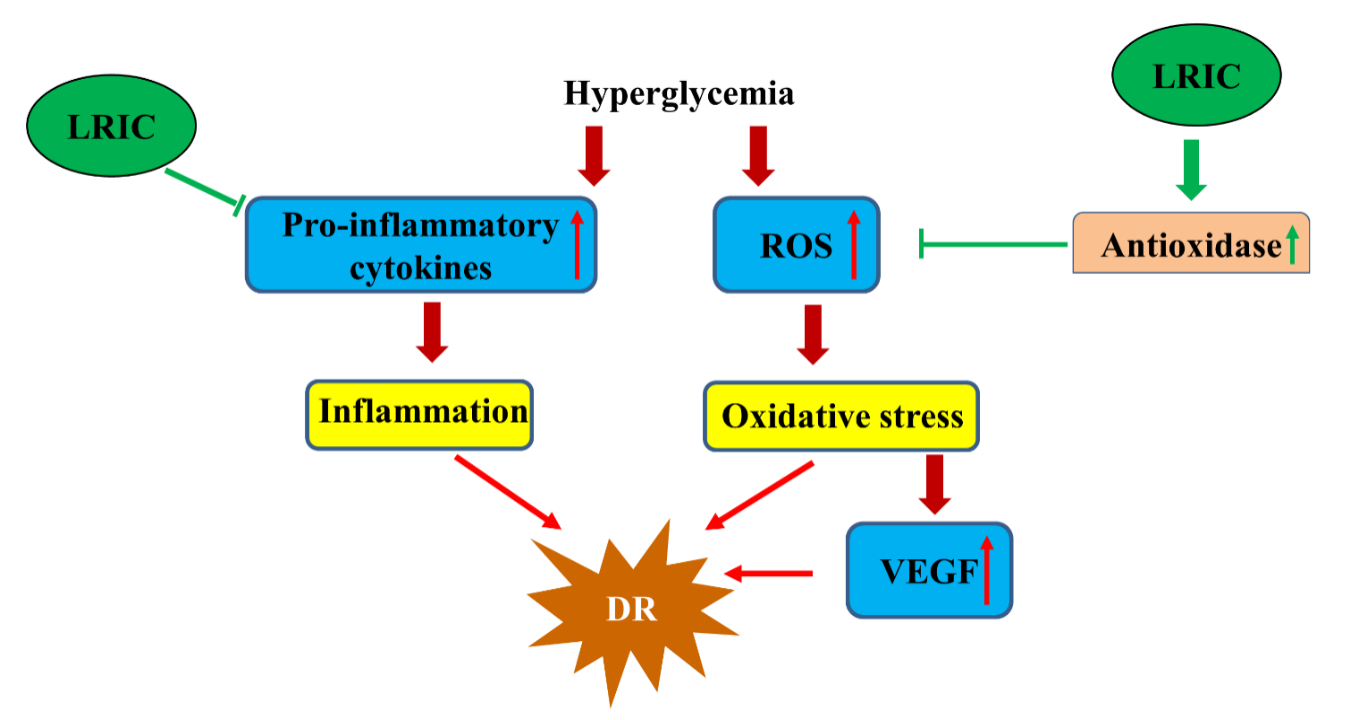
Hypothesis regarding the mechanism of LRIC’s protective effects against diabetic retinopathy.

In this study, we demonstrated that LRIC had a neuroprotective effect over diabetic retinopathy. LRIC treatment ameliorated Brn3a+ retinal ganglion cell loss and attenuated retinal Müller cell activation. LRIC reduced inflammatory cytokine (IL-6) and ROS levels as well as increase the levels of antioxidants. Taken together, this study suggests that LRIC could exert remarkable neuroprotective effects against diabetes-induced RGC degeneration in rats by attenuating the progression of diabetic retinopathy through anti-inflammatory and antioxidative mechanisms (Fig. 8).

Our results showed that LRIC had no effect on blood glucose. We speculated that the protective mechanism of LRIC on DR is not through a reduction of blood glucose level. Although diabetic patients receive intensive glycemic control (median HbA1c: 7.3%) with conventional (median HbA1c: 9.1%) treatment, 24% patients still develop DR [3]. We therefore suggest that LRIC be used as an adjunct therapy to delay the progression of DR.

Diabetic rodents exhibited progressive loss of RGCs in the ganglion cell layer during diabetes [14]. A reliable measure of RGC is of importance to evaluate the efficacy of RGC degenerative diseases [23]. The Brn3 family of POU-domain transcription factors plays an important role in differentiation and survival during the development of murine RGCs, thus indicating that Brn3a is a specific and reliable marker for identifying RGCs [24, 25]. Using Brn3a immunohistochemical staining, we found that after 12 weeks from the onset of diabetes, the number of RGCs was reduced by approximately 17% compared with the normal control group. Our results showed that LRIC treatment attenuated the loss of RGCs. The neuroprotective effect of LRIC in the retina is consistent with our previously published observations [17]. The loss of RGC bodies is also reflected by a reduction in the number of axons in the optic nerve [26]. In a group of people with 15 years of diabetes, the thickness of the nerve fiber layer in the superior polar quadrant of the retina was significantly reduced compared with the control group [26, 27]. Although LRIC treatment had no effect in reducing blood glucose level, special attention should still be given to its role in neuroprotection.

Reactive gliosis is one of the early features of the diabetic retina. Müller cells and astrocytes undergo activation in diabetes, and GFAP expression in Müller cells is widely considered to be a sensitive marker of retinal stress [28]. In the normal control retina, we found that GFAP staining was only restricted to astrocytes and not found in Müller cells. Consistent with previous studies, our results showed that GFAP is up-regulated in STZ-induced diabetic rats compared with normal control rats. After LRIC treatment, GFAP expression is decreased compared to untreated diabetic retinas, but remained higher than that in normal control retinas, indicating that LRIC inhibited Müller cell activation in diabetic rats. A similar result was observed in Fernandez’s study. They showed that retinal ischemic conditioning also reduced GFAP immunoreactivity [12]. In fact, results from this study are also consistent with our previous report where LRIC reduced GFAP immunoreactivity in a retinal ischemic rat model.

It is well known that oxidative stress is an important factor in the pathology of DR. Persistent hyperglycemia induces ROS formation and triggers oxidative stress. ROS injures retinal endothelial cells by damaging proteins, lipids, and DNA [8]. In this study, we found that STZ-induced diabetic rats exhibited a higher level of ROS compared with normal control rats; LRIC treatment significantly reduced the ROS level. Excessive ROS in the retina are mainly scavenged by antioxidant enzymes such as SOD and peroxidase [29]. Our results showed that LRIC markedly increased SOD and SOD/CAT ratio as w as GSH/GSSG ratio, an index of total tissue antioxidant capacity. Our recent report showed that LRIC was able to increase SOD activity in a cerebral ischemia-reperfusion rat model [30]. Elevated inflammatory reaction also plays important roles in the pathogenesis of DR [31]. It has been reported that there were increases in the levels of pro-inflammatory cytokines including IL-1β, IL-6 and TNF-α in retinal tissues of animals and patients with DR [32, 33]. In this study, we found that the levels of IL-1β, IL-6 and TNF-α were increased in the retinal tissue in DM only rats compared with normal control rats. LRIC treatment significantly reduced the level of IL-6 both in the retinal tissue and plasma. This is consistent with our previous study, daily LRIC treatment reduced IL-6 plasma level in intracranial arterial stenosis patients [16]. Taken together, these observations strongly suggest that LRIC is a potential therapeutic against oxidative stress and inflammation.ell

Intraocular VEGF expression is reported to be upregulated in diabetic patients and animals [34, 35]. The increased VEGF expression triggers leukocyte aggregation, and inflammatory response [36]. This inflammation in turn stimulates a further release of VEGF [37, 38]. Our present study showed that the expression of VEGF significantly increased in the DR rats compared with normal control rats. However, LRIC treatment significantly decreased the expression of VEGF. Contrary to our experimental results, some studies showed that ischemia or hypoxia conditioning were able to increase VEGF expression [39, 40]. We then speculated that the reason for this discrepancy may be related to the disease model and the application method of LRIC. On the other hand, LRIC, an endogenous protection mechanism, could have bidirectional regulation of which remains unknown. The ischemic conditioning stimulus can be applied before ischemia (pre-conditioning), during ischemia (per-conditioning), or during reperfusion after ischemia (post-conditioning) [41]. Fernandez’s study found that b waves decreased at 6 weeks of hyperglycemia and further decreased at 10 weeks. Weekly application of 5 min retinal ischemia conditioning, which was induced by a transient period of brief high intraocular pressure, significantly prevented the decreased of a- and b- wave amplitude. Furthermore, delayed treatment started at 6 weeks also protected the diabetic retina from functional alteration [12] although there were no observable changes in RGCs even at 10 weeks after diabetes onset. Possible explanation for this is that functional changes may precede morphological changes in the diabetic retina. Salido’s study demonstrated that retinal ischemic preconditioning was able to protect Type 2 diabetic retinopathy by reducing retinal lipid peroxidation, NOS activity, TNFα and VEGF levels while increasing catalase activity [13]. Research showed that axonal changes at the distal portion of the optic nerve could be the first structural change in the early stages of diabetic rats [42]. However, the retina is susceptible to ischemic injury. Compared to retinal conditioning, remote ischemic conditioning is safe, feasible, and reliable and could easily be translated to the clinic [43]. Our previous study have showed that ischemic postconditioning protected RGCs from ischemia insult at day 3 and day 7 following reperfusion, decreased GFAP expression and increased the expression of Nrf2 and HO-1 [17]. These findings in our study indicate that LRIC techniques may be a potential therapy for the treatment of DR in clinic.

### Conclusion

LRIC ameliorates ganglion cell loss and ameliorated retinal Müller cell activation in STZ-induced diabetic rats. LRIC enhances the activities of SOD and GSH, increases GSH/GSSG ratio and inhibits the production of ROS, IL-6, and VEGF. These results suggest that the antioxidative and anti-inflammatory activities of LRIC may be important mechanisms involved in the protective effect of LRIC on DR in STZ-induced diabetic rats.
